# Effect of active wound dressing on postoperative pain and wound healing in patients undergoing anorectal surgery

**DOI:** 10.1186/s12876-025-03922-y

**Published:** 2025-04-30

**Authors:** Guangyuan Qie, Miao Wan, Mengdie Cui, Wen Xia, Qianqian Liu

**Affiliations:** 1 General Medicine Department, The First Affiliated Hospital, Naval Military Medical University, Shanghai, 200433 China; 2 Department of Colorectal Surgery, The First Affiliated Hospital, Naval Military Medical University, Shanghai, 200433 China

**Keywords:** Active wound dressing, Anorectal surgery, Postoperative pain, Wound healing

## Abstract

**Aim:**

To study the effect of active wound dressing on postoperative pain and wound healing in patients undergoing anorectal surgery (AS).

**Methods:**

The clinical data of 110 AS patients from March 2023 to January 2024 were retrospectively screened. The study group (SG) included 55 patients who received active wound dressing post-operation, while the control group (CG) consisted of 55 patients who received conventional dressing, matched for baseline indices. Postoperative pain degree, wound healing rate, healing time, swelling disappearance time, dressing change times, granulation tissue formation time, growth factor levels, and inflammatory factor levels were compared between the groups.

**Results:**

There were notable variations in the time effect, inter-group effect and time × inter-group effect in the postoperative pain score and wound healing rate between the two groups (*P* < 0.05). The pain scores of the SG on the 1st, 3rd and 7th day after operation were lower (*P* < 0.05). The wound healing rate of the SG was higher on the 1st, 3rd and 7th day after operation (*P* < 0.05). The wound healing time, wound swelling disappearance time, dressing change times and granulation tissue formation time in the SG were shorter (*P* < 0.05). The levels of vascular endothelial growth factor, epidermal growth factor and basic fibroblast growth factor in the SG were higher (*P* < 0.05). The levels of IL -8 and TNF-α in the SG were lower (*P* < 0.05).

**Conclusion:**

Active wound dressing has been shown to alleviate postoperative pain in patients undergoing AS, promote wound healing, and effectively regulate levels of growth factors and inflammatory factors.

## Introduction

Anorectal surgery (AS) is a common surgical method to treat hemorrhoids, anal fissure, rectal prolapse and other diseases, with a high success rate. Because of the sensitivity and complexity of the surgical site, postoperative pain management and wound healing have always been the focus of attention of both doctors and patients [1, 2]. While traditional wound treatment methods meet basic needs to some extent, there remains significant room for improvement in enhancing healing efficiency and alleviating patient pain. Therefore, finding effective pain management strategies and methods to promote wound healing is crucial for improving patient prognosis. Active wound dressing, featuring unique bioactive components and intelligent design, surpasses traditional dressings’ limitations, such as limited absorption capacity, poor air permeability, and increased infection risk. It provides a more conducive healing environment by effectively absorbing secretions, maintaining wound moisture, and stimulating cell proliferation, angiogenesis, and tissue remodeling through the release of specific factors, thereby accelerating wound healing [3]. Active wound dressing can effectively reduce pain, promote wound healing, and minimize scar formation in second-degree or third-degree burns and scalds. Additionally, it aids in debriding necrotic tissue, stimulating new tissue growth, and accelerating healing in chronic ulcerative conditions such as diabetic foot ulcers and pressure sores [4–6]. While active wound dressing demonstrates significant potential in various conditions mentioned, its efficacy in AS patients requires further clinical validation. This study aims to evaluate the efficacy of commercially available active wound dressings in anorectal surgery recovery to provide evidence-based data for their application in routine clinical practice. Unlike previous studies that focused on general wound management, this study uniquely targets the specialized healing challenges presented in anorectal surgical wounds. Thus, the purpose of this research is to assess the application of active wound dressing post-anorectal surgery, specifically assessing its impact on postoperative pain management and wound healing speed. The goal is to provide clinicians with valuable insights to optimize postoperative recovery for patients.

## Materials and methods

### Sample size calculation

The formula below is used to determine the sample size: The rate is estimated and calculated with the hypothesis test formula “n = Z²α/2π (1-π) /δ²”. When the class I risk probability α is 0.05, the class II risk probability β is 0.10, Z²α/2 = 11, π is 0.10, the maximum sample size can be obtained to ensure the accuracy of the study, and the allowable error (δ) is 0.03, then the calculated n is 1100. The sample size was reduced by 90%, and 110 cases were finally included.

### Inclusion criteria and exclusion criteria

Inclusion criteria: (1) Patients with anal fissure, anal fistula, and Prolapse of rectum and haemorrhoids, conforming to the relevant diagnostic criteria outlined in the Clinical Practice Guidelines for anorectal daytime surgery (2019 edition) [7]; (2) All patients underwent AS, including I grade and II grade; (3) Postoperative signs stable and clear consciousness; (4) Age over 18 years; (5) Good compliance and full clinical information. Exclusion criteria: (1) Combined with malignant tumors, immune system disorders, or blood disorders; (2) Infectious diseases involving the gastrointestinal tract; (3) History of allergic constitution; (4) Pregnant or lactating female patients; (5) Concurrent mental illness; (6) Complicated with severe diseases such as cardiovascular, neurological, hepatic, or renal disorders, or chronic conditions like hypertension and diabetes.

### General information

The clinical data of 110 patients undergoing AS from March 2023 to January 2024 in our hospital were shown in the past. Based on several techniques for treating wounds, individuals who received active wound dressing post-operation (*n* = 55) were included in the study group (SG), while individuals who received conventional dressing post-operation, matched for baseline indices (*n* = 55), were included in the control group (CG). Every patient signed the informed consent form and gave their approval. This research has been reviewed by our hospital’s Medical Ethics Committee and complies with the Helsinki Declaration (approval number NMH202403056). The SG and the CG do not vary statistically (*P* > 0.05, Table 1).

### Methods

Commercially available alginate dressings were selected to align with real-world clinical practices, ensuring the study’s findings could be readily implemented in routine medical settings. This choice allows for broader reproducibility across healthcare institutions. Both groups underwent conventional potassium permanganate solution sitz baths (1/1000 concentration, 20 min, twice daily), regular dressing changes, and observation of wound conditions. SG and CG groups were matched based on age, comorbidities, and the severity of the surgical condition using propensity score matching. This approach was chosen to minimize the impact of potential confounders and ensure comparability between the groups. The CG received conventional dressing post-sitz bath, using sterilized Vaseline gauze (Zhejiang Aoqi Medical Dressing Co., LTD.) applied to the surgical wound and secured with medical tape. The SG was given active wound dressing, self-adhesive alginate dressing [Kelu (Wuhan) Biotechnology Co., LTD., (80–300) mm× (80–300) mm], external wound dressing. In both groups, wound dressings were changed twice daily during the first 7 days postoperatively, followed by once daily until complete epithelialization was achieved.

### Surgical procedures

All patients underwent standardized procedures: lateral internal sphincterotomy for anal fissures, fistulotomy/seton for anal fistulas, Milligan-Morgan hemorrhoidectomy for hemorrhoids, and Delorme’s or Altemeier procedure for rectal prolapse. All operations were performed under spinal anesthesia in the jackknife position.

### Observation index

The postoperative pain degree, wound healing rate, wound healing time, wound swelling disappearance time, dressing change times, granulation tissue formation time, growth factor level and inflammatory factor level of the two groups were evaluated.

**Fig. 1:**
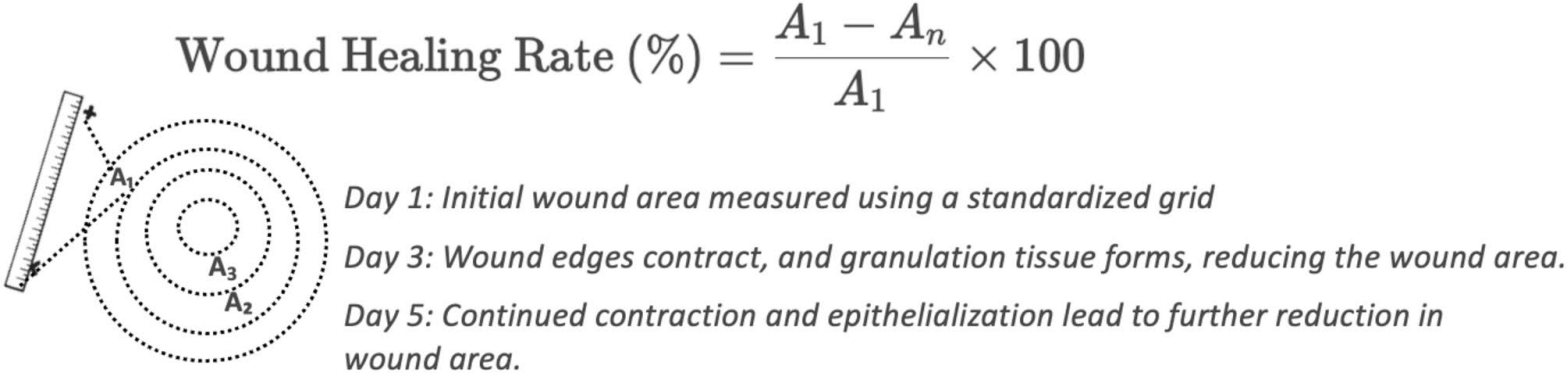
wound healing rate measurement process

(1) Postoperative pain degree: visual analogue scale [8] was employed to assess the patients’ pain on the 1st, 3rd, and 7th day after operation, with a total score of 10, with 0 being painless and 10 being the most painful.

(2) For patients, the wound healing rate was determined using the following formula on the first, third, and seventh days after surgery: wound healing rate = (original area - current wound area) / original area × 100%. A diagram shown in Fig. 1.

(3) Wound healing parameters included observing and recording the time to complete wound healing, disappearance of wound swelling, number of dressing changes, and time to formation of granulation tissue. Granulation tissue formation time was assessed using a standardized visual inspection protocol, supported by photographic evidence. Two independent observers evaluated the wounds to ensure consistency and accuracy in the assessment.

(4) Growth factor levels were assessed by collecting 5 mL of venous blood in both SG and CG group before and after the intervention (at 0 days and 7 days). The serum was extracted from the blood samples and frozen for analysis after they were centrifuged for ten minutes at 3000 rpm. Using the enzyme-linked immunosorbent assay (ELISA) and an automated biochemical analyzer (Mindrays, BS-400) supplied by Kelu (Wuhan) Biotechnology Co., LTD, the levels of vascular endothelial growth factor (VEGF), epidermal growth factor (EGF), and basic fibroblast growth factor (bFGF) were measured. These tests were part of the routine postoperative monitoring protocol in our hospital, and the data were retrospectively collected from patient records to ensure consistency between the study group and the control group.

(5) Inflammatory factors (TNF-α and IL-8) were measured in both SG and CG groups before and after the intervention (at 0 days and 7 days). Blood samples (5 mL) were collected from all patients, and serum was extracted after centrifugation at 3000 rpm for 10 min. Tumor necrosis factor-α (TNF-α) and interleukin-8 (IL-8) levels were assessed using an automated biochemical analyzer and the enzyme-linked immunosorbent assay (ELISA). The instrument model and the manufacturer of the ELISA kits were identical to those previously mentioned.

### Flow chart

**Fig. 2:**
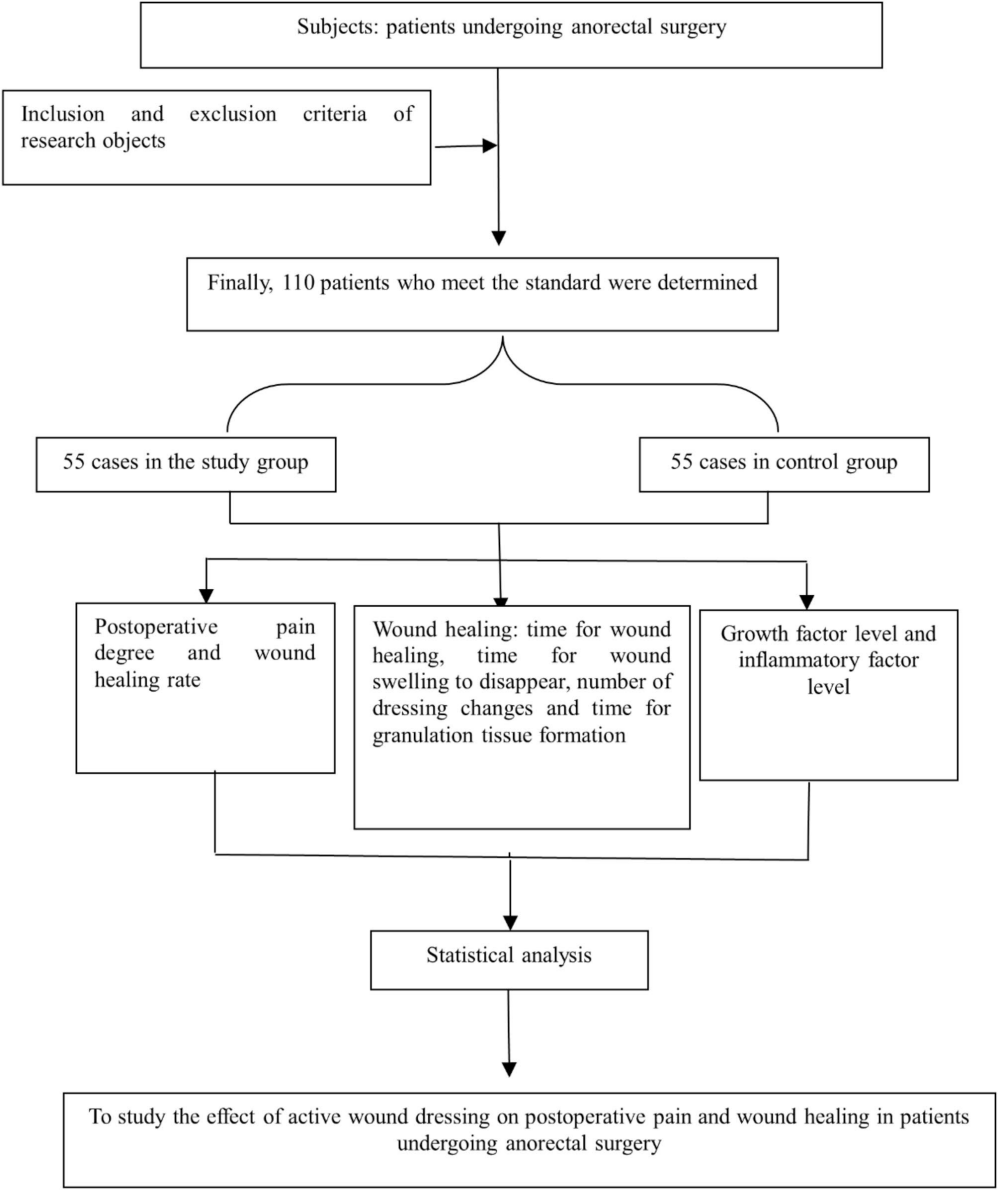
Research flow chart

Figure 2 shows the flow chart of the research.

### Statistical methods

Software SPSS26.0 was used for statistical analysis, while Prism 9.4.1 was utilized to analyze the photos. The measurement data, which were represented by ($\bar{x}\pm\:s$), fit the normal distribution. ANOVA with repeated measurements was used at various intervals. The count data were reported as a percentage, and the comparison was carried out using the χ2 test. The pairwise comparison was carried out using the least significant difference (LSD)-t test, or t test. Statistics were deemed significant if *P* < 0.05.

## Results

### Comparison of two groups of general data

There was not a discernible distinction between the SG and the CG in terms of general data (*P* > 0.05), as seen in Table 1.

**Table 1:** Comparison of two groups of general data (*n* = 55)

Group	Gender		Age (years)	Course of disease (months)	Operation time(mins)	ASA grade		Suffer from a disease			
	Male	Female				I grade	II grade	anal fistula	anal fissure	Haemorrhoids	Prolapse of rectum
Study group	31(56.36)	24(43.64)	47.69 ± 5.23	5.23 ± 0.63	72.38 ± 9.06	33(60.00)	22(40.00)	11(20.00)	18(32.73)	23(41.82)	3(5.45)
Control group	33(60.00)	22(40.00)	47.73 ± 5.25	5.20 ± 0.61	72.44 ± 9.09	38(69.09)	17(30.91)	10(18.18)	17(30.91)	21(38.18)	7(12.73)
*χ*^2^/*t*	0.149		0.040	0.253	0.034	0.993		1.767			
*P*	0.699		0.968	0.800	0.972	0.319		0.622			

### Comparison of postoperative pain between the two groups

Analysis of variance of repeated measurement: There were significant differences in time effect, inter-group effect and time × inter-group effect involving the two groupings (*P* < 0.05). Pairwise comparison and analysis: the pain scores of the two groupings were lower on the 7th postoperative day than on the 1st and 3rd postoperative day, and the pain scores of the SG were lower on the 1st, 3rd, and 7th postoperative day (*P* < 0.05), as seen in Table 2.

**Table 2:** Comparison of postoperative pain involving the two groupings (*n* = 55, points, $\bar{x}\pm\:s$)

Group	1 day after operation	3 days after operation	7 days after operation
Study group	7.13 ± 0.71	5.81 ± 0.64^①^	2.37 ± 0.26^①^^②^
Control group	8.15 ± 0.83	6.79 ± 0.71^①^	4.05 ± 0.35^①^^②^
*LSD-t*	6.925	7.603	28.575
*P*	< 0.05	< 0.05	< 0.05
*F*_*time*_ */P*	81.562/<0.05		
*F*_*interblock*_ */P*	8.917/<0.05		
*F*_*time × interblock*_ */P*	1.294/0.05		

Note: ① Compared with 1 day after operation in the group, *P* < 0.05; ② Compared with postoperative 3 days in the group, *P* < 0.05

### Comparison of wound healing rate between two groups

**Fig. 3:**
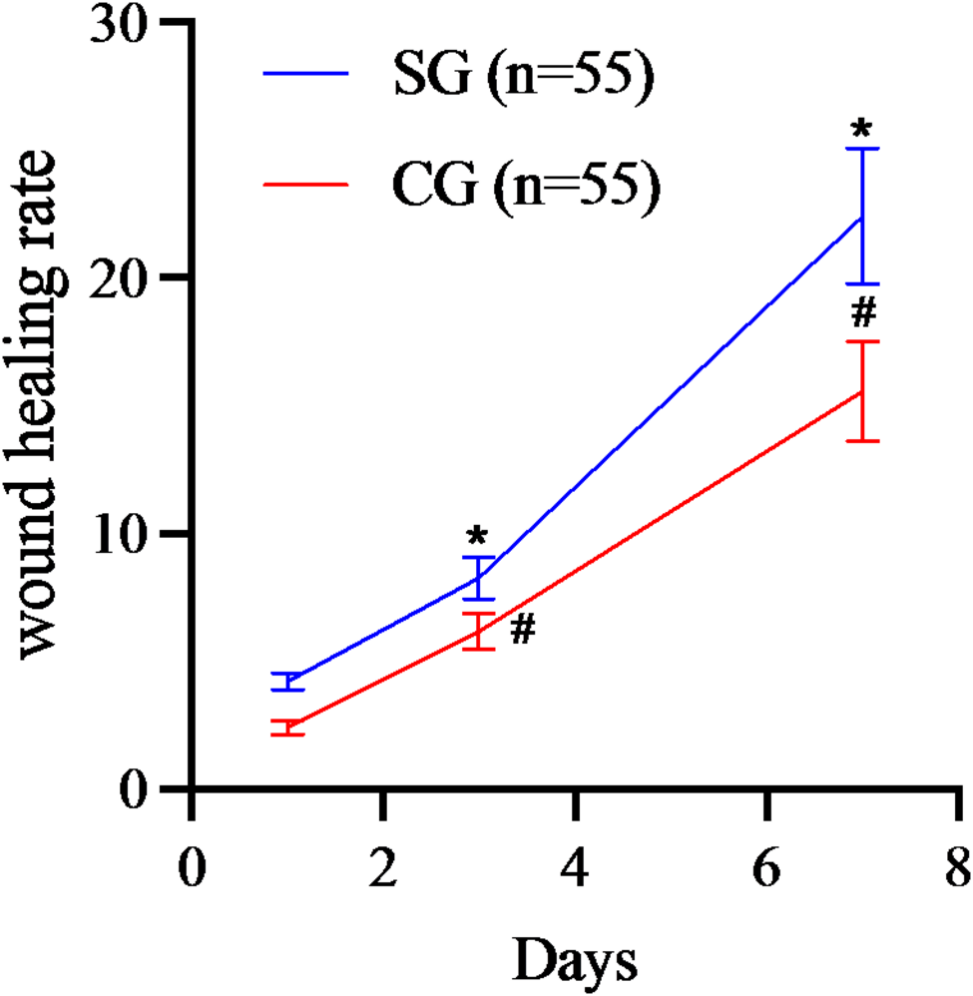
Comparison of wound healing rate involving two groupings (SG,^*^P < 0.05, CG,^#^*P* < 0.05)

Repeated measures analysis of variance revealed statistically significant differences in the time effect, inter-group effect, and time × inter-group effect of postoperative wound healing rates involving the two groupings (*P* < 0.05). Pairwise comparison analysis showed that the wound healing rate on day 7 post-surgery was higher than on days 1 and 3 post-surgery for both groups. Additionally, the wound healing rate on day 3 post-surgery was higher than on day 1 post-surgery for both groups. Furthermore, the SG exhibited higher wound healing rates than the CG on days 1, 3, and 7 post-surgery (*P* < 0.05, Table 3; Fig. 3).

**Table 3:** Comparison of wound healing rate involving two groupings (*n* = 55, %, $\bar{x}\pm\:s$)

Group	1 day after operation	3 days after operation	7 days after operation
Study group	4.22 ± 0.33	8.25 ± 0.85^①^	22.41 ± 2.26^①^^②^
Control group	2.42 ± 0.28	6.17 ± 0.72^①^	15.55 ± 1.95^①^^②^
*LSD-t*	30.845	13.847	17.043
*P*	< 0.05	< 0.05	< 0.05
*F*_*time*_ */P*	87.650/<0.05		
*F*_*interblock*_ */P*	6.336/<0.05		
*F*_*Time ×interbloc*_ */P*	2.666/<0.05		

Note: ① Compared with 1d after operation in the group, *P* < 0.05; ② Compared with postoperative 3 days in the group, *P* < 0.05

### Comparison of wound healing between the two groups

The wound healing time, wound swelling disappearance time, dressing change times and granulation tissue formation time in the SG were all shorter (*P* < 0.05, Table 4).

**Table 4:** Comparison of wound healing involving the two groupings (*n* = 55, $\bar{x}\pm\:s$)

Group	Wound healing time(d)	Disappearing time of wound swelling(d)	Number of dressing changes (times)	Granulation tissue formation time(d)
Study group	15.44 ± 1.69	9.21 ± 1.01	10.55 ± 1.16	3.27 ± 0.36
Control group	18.27 ± 1.73	10.46 ± 1.07	12.67 ± 1.23	4.63 ± 0.41
*T*	8.678	6.300	9.299	18.485
*P*	< 0.05	< 0.05	< 0.05	< 0.05

### Comparison of growth factor levels involving the two groupings

There wasn’t a noticeable variation in growth factor levels involving the two groupings before intervention (*P* > 0.05). After the intervention, the levels of growth factors in both groups increased, and the levels in the SG were higher than those in the CG (*P* < 0.05, Table 5).

**Table 5:** Comparison of growth factor levels between the two groups (*n* = 55, $\bar{x}\pm\:s$)

Group	Vascular endothelial growth factor(ng/L)		Epidermal growth factor(ng/L)		Basic fibroblast growth factor(µg/L)	
	BI	AI	BI	AI	BI	AI
Study group	80.55 ± 3.89	97.65 ± 4.12^③^	460.86 ± 13.25	566.47 ± 10.98^③^	36.53 ± 4.39	56.46 ± 5.47^③^
Control group	80.58 ± 3.92	93.24 ± 4.03^③^	461.91 ± 13.06	537.59 ± 14.34^③^	36.58 ± 4.43	51.93 ± 5.56^③^
*T*	0.040	5.674	0 418	11.858	0.059	4.307
*P*	0.967	< 0.05	0.676	< 0.05	0.952	< 0.05

Note: ③ Compared with before intervention in the group, *P* < 0.05. BI indicates Before intervention. AI indicates after intervention

### Comparison of IF involving the two groupings

**Fig. 4:**
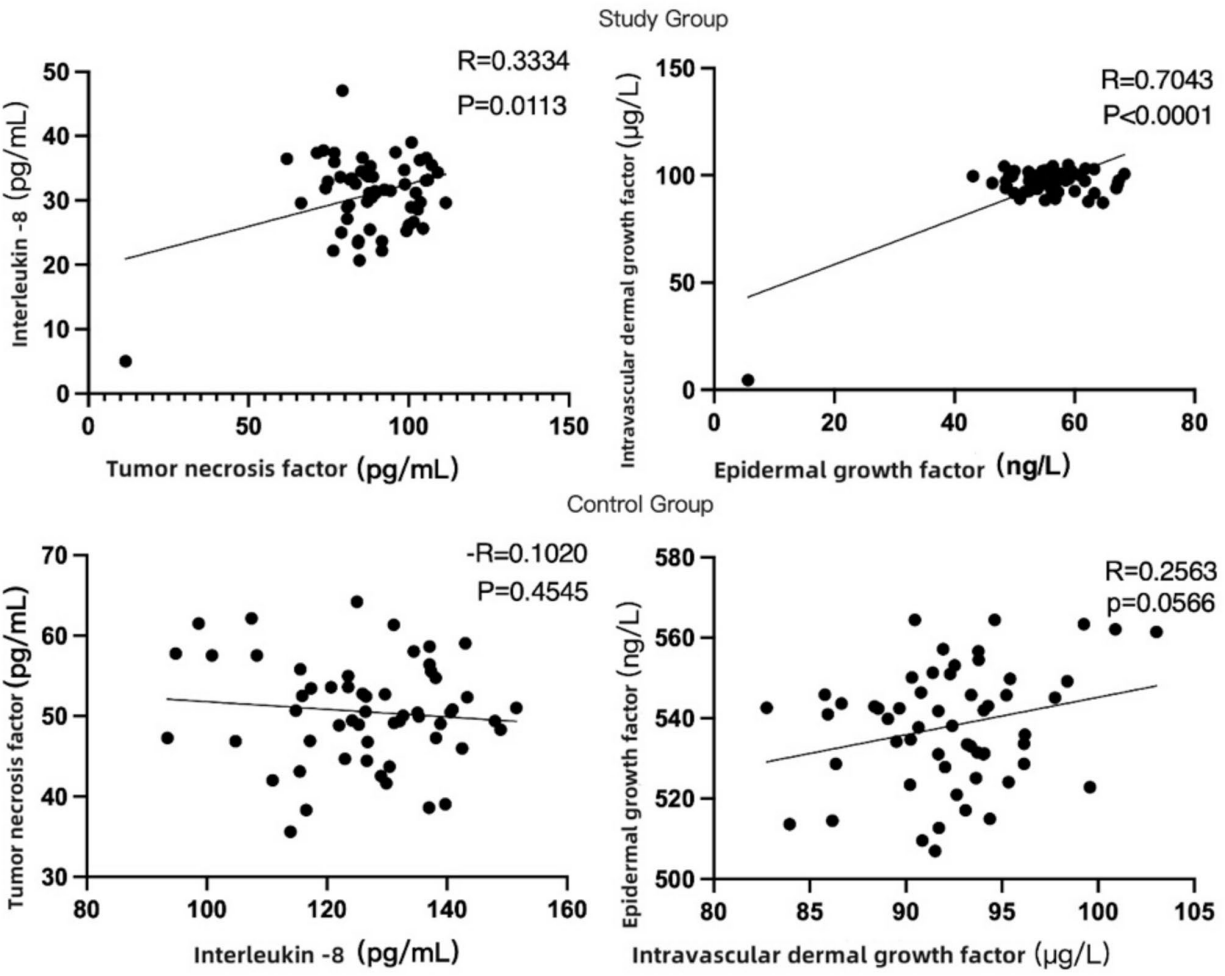
Correlation analysis of IF and growth factors

There wasn’t a noticeable variation in the levels of IF involving the two groupings before intervention (*P* > 0.05). After the intervention, the levels of IF in both groups decreased, and the levels in the SG were lower (*P* < 0.05, Table 6). In the SG after intervention, the correlation coefficient of IF was *R* = 0.3334, *P* = 0.0113, indicating a positive correlation that was statistically significant. Additionally, the correlation coefficient between epidermal growth factor (EGF) and vascular endothelial growth factor (VEGF) was *R* = 0.7043, *P* < 0.0001, demonstrating a strong positive correlation that was highly significant. In contrast, there was no discernible relationship between the levels of IF and growth factors in the CG (Fig. 4). Both groups experienced no complications or infections.

**Table 6:** Comparison of levels of IF involving two groupings (*n* = 55, $\bar{x}\pm\:s$)

Group	Interleukin-8(IL-8,pg/mL)		Tumor necrosis factor-α(pg/mL)	
	BI	AI	BI	AI
Study group	245.36 ± 14.35	86.54 ± 12.16^③^	93.14 ± 13.27	31.05 ± 5.64^③^
Control group	244.27 ± 16.58	125.45 ± 14.37^③^	94.23 ± 11.09	49.86 ± 6.75^③^
*T*	0.368	15.329	0.467	15.859
*P*	0.713	< 0.05	0.641	< 0.05

Note: ③ Compared with before intervention in the group, *P* < 0.05. BI indicates Before intervention. AI indicates after intervention

## Discussion

After AS, patients often encounter issues such as postoperative pain and delayed wound healing, which not only heighten discomfort but can also extend recovery periods and impact quality of life. Active wound dressings typically incorporate bioactive components like growth factors, antibacterial agents, and moisturizers. These components aid in the rapid removal of wound exudate, reduce infection risks, and facilitate the formation of new tissue, thereby accelerating the wound healing process [9]. Some studies have found that the use of specific types of active dressings can significantly reduce postoperative pain, reduce bleeding and exudation, and improve wound healing efficiency [10, 11]. While existing research has yielded some advancements, further verification is needed to assess the efficacy of active wound dressing in helping people with AS manage their pain after surgery and encourage the healing of their wounds. While the use of a commercially available product limits exploration of novel material properties, the focus of this study was to evaluate clinical efficacy rather than material innovation. Future research could explore custom-designed dressings to bridge this gap.

The study results demonstrated that the pain scores of the SG were considerably lower on the 1st, 3rd, and 7th days after surgery, indicating that active wound dressing effectively alleviates postoperative pain in patients undergoing AS. This finding aligns with the findings of Wen Xu et al. [12], who reported that nano-silver antibacterial hydrogel dressing reduced pain following debridement of ankle open fractures. Active wound dressings typically contain calcium alginate and various bioactive components that promote cell growth and wound healing acceleration. They absorb wound secretions, maintain a dry wound environment, and minimize discomfort caused by moisture. Additionally, active wound dressings create a protective barrier against external stimuli, further reducing pain. Studies have shown that alginate dressings can form a stable gel on the wound surface of patients with anal fistulas, providing an optimal moist environment without toxic side effects or irritation to the wound surface [13]. Therefore, the pain-relieving effects of active wound dressings may be attributed to their ability to create a protective barrier, maintain a moist wound environment, and reduce mechanical irritation. Additionally, the bioactive components of the dressings, such as alginate and silver ions, may interact with nerve endings to modulate pain perception [14].

The study results revealed that the wound healing rate in the SG was considerably higher on the 1st, 3rd, and 7th days after surgery. Additionally, the SG exhibited shorter wound healing times, quicker disappearance of wound swelling, fewer dressing changes, and faster granulation tissue formation compared to the CG. These findings suggest that active wound dressing promotes postoperative wound healing in patients undergoing AS and enhances wound healing speed. Previous research has indicated that maintaining a moist wound environment facilitates cell migration, proliferation, epithelialization, and granulation tissue formation, thereby accelerating the overall healing process [15]. In the process of wound healing, neovascularization is very important [16]. The growth factors included in active dressings encourage vascular endothelial cells to proliferate and migrate, which aids in the development of new blood vessels. This process ensures adequate oxygen and nutrient supply to wounds. Additionally, these components foster the proliferation and differentiation of fibroblasts and keratinocytes, thereby accelerating wound repair. Infection stands out as a primary factor contributing to delayed wound healing [17]. The antibacterial properties of active wound dressings lower the chance of infection and enhance the healing of wounds. In a randomized controlled trial comparing active dressing with traditional dressing, patients using active dressing demonstrated significant advantages in both wound healing time and quality [18]. In addition, a number of systematic reviews and meta-analyses also confirmed the positive role of active dressings in improving the healing rate and reducing complications [19, 20].

The results of this study indicated higher levels of basic fibroblast growth factor (bFGF), EGF and VEGF in the SG, recommending that active wound dressing effectively regulates these growth factors in patients undergoing AS. VEGF plays a crucial role in promoting angiogenesis, which is essential for vascular reconstruction during wound healing [21]. The components within active wound dressings stimulate the production of VEGF, increasing local vascular density in the wound. This process enhances oxygen and nutrient supply, accelerating wound healing. Previous studies have similarly demonstrated these effects. Compared with the traditional dressing, the active dressing containing vascular endothelial growth factor can significantly shorten the wound healing time of patients [22]. EGF is an important mitogen, which can stimulate the proliferation of many kinds of cells, especially epidermal cells [23]. Active wound dressing accelerates the migration and proliferation of wound epithelial cells by increasing epidermal growth factor, thereby expediting wound closure. International studies have highlighted that utilizing active dressings containing epidermal growth factor significantly enhances the healing rate of chronic wounds and reduces healing time [24]. The bFGF is a potent mitogen, which has a strong proliferative effect on fibroblasts, vascular endothelial cells and smooth muscle cells [25]. Active wound dressing promotes granulation tissue formation and accelerates wound repair by enhancing basic fibroblast growth factor. In a study involving patients undergoing AS, those using active dressings containing basic fibroblast growth factor demonstrated significantly shorter wound healing times and higher wound strength post-healing compared to those using traditional dressings [26]. Furthermore, a research confirmed the growth factors present in active wound dressings, not only promote tissue repair but may also play a role in regulating the inflammatory response. These factors can influence the release of pro-inflammatory cytokines, thereby modulating the inflammatory environment and promoting faster healing [27].

The study’s findings also demonstrated that the levels of TNF-α and IL-8 in the SG were lower, suggesting that active wound dressing can reduce the postoperative inflammatory reaction of patients undergoing AS. TNF-α is a key pro-inflammatory cytokine that can delay wound healing if present in excessive amounts, while IL-8 is involved in neutrophil recruitment and angiogenesis. By measuring these markers, we were able to evaluate the impact of active wound dressings on the inflammatory response and its subsequent effect on wound healing [28, 29]. The effective sealing and adhesion of sodium alginate fibers in active wound dressings reduce bacterial load on the wound surface. Additionally, the presence of silver ions in these dressings helps alleviate local inflammatory reactions. Previous studies have shown that silver ion release dressing has been proved to effectively reduce the growth of Staphylococcus aureus and Escherichia coli, and then reduce the secretion of TNF-α and IL-8 [30, 31]. Active dressing contains growth factors or extracellular matrix analogues, which can stimulate the proliferation of fibroblasts and endothelial cells and accelerate the epithelization and healing process of wound surface. A randomized controlled trial found that the level of postoperative IF in surgical patients using silver ion release dressing was significantly lower than that in patients using traditional dressing [32]. Active dressings containing growth factors show a lower level of IF in the treatment of wound infection than standard treatment, which indicates that such dressings may have potential advantages in regulating inflammatory response [33, 34]. Furthermore, dressings containing silver ions have demonstrated efficacy against bacterial biofilms [35]. Strong antibacterial action is shown by seaweed fibers treated with sodium alginate and silver ions against Escherichia coli and Staphylococcus aureus, with antibacterial rates over 99% for both pathogens [36].

Therefore, active wound dressing can effectively mitigate the inflammatory reactions in patients undergoing AS through its unique bioactive components and mechanisms, thereby promoting wound healing and lowering the risk of postoperative complications. The findings of this study have important implications for clinical practice, particularly in the management of postoperative pain and wound healing in anorectal surgery. Active wound dressings could be integrated into postoperative care protocols to improve patient outcomes and reduce recovery times. Future research should focus on the cost-effectiveness and practical implementation of these dressings in routine clinical settings.

Moreover, our study has several limitations. Firstly, the retrospective design may introduce selection bias, despite our efforts to match the groups based on baseline characteristics. Randomized controlled trials (RCTs) are considered the gold standard for establishing causality, and our future studies will consider a prospective RCT design to reduce potential biases and provide stronger evidence of the efficacy of active wound dressings. Secondly, in many Western countries, anorectal surgeries are often performed on a day-surgery basis, which may limit the feasibility of using active dressings due to cost and logistical constraints. Third, as our study focused on anorectal procedures with clinically significant external wounds, the findings may not fully generalize to closed surgical approaches (e.g., stapled hemorrhoidopexy or mucosal-only prolapse repairs). While this design ensured standardized wound assessment, it highlights the need for future investigations to: (1) establish procedure-specific wound care algorithms based on surgical trauma extent, and (2) develop biomarkers to objectively quantify ‘invisible’ mucosal healing where dressings are not applicable. While our study demonstrates the efficacy of active wound dressings in a Chinese healthcare setting, further research is needed to evaluate their applicability in Western healthcare systems, particularly in the context of day-surgery protocols. Future studies should consider the cost-effectiveness and logistical feasibility of implementing active dressings in different clinical settings. Future prospective, multicenter studies with larger sample sizes are needed to confirm our results. Despite these limitations, our study provides valuable insights into the efficacy of active wound dressings in anorectal surgery, particularly in reducing postoperative pain and promoting wound healing.

## Conclusion

In summary, active wound dressing demonstrates potential in relieving postoperative pain, promoting wound healing, and effectively regulating levels of growth factors and IF in patients undergoing AS. This study’s limitations include its limited sample size and single-center methodology. The study did not stratify groups based on disease type, age, or sex, which may influence postoperative pain and wound healing outcomes. To further confirm these results, multicenter studies with bigger sample numbers should be a part of future study. The findings of this study have important implications for clinical practice, particularly in the management of postoperative pain and wound healing in anorectal surgery. Active wound dressings could be integrated into postoperative care protocols to improve patient outcomes and reduce recovery times. Future research should focus on the cost-effectiveness and practical implementation of these dressings in routine clinical settings.

## Data Availability

The datasets used and/or analyzed during the current study are available from the corresponding author on reasonable request.
